# The Use of Point-of-Care Ultrasound for a Rapid Diagnosis of Endophthalmitis: A Case Report

**DOI:** 10.7759/cureus.68609

**Published:** 2024-09-04

**Authors:** Varsha S Shinde, Joshua D Birru

**Affiliations:** 1 Emergency Medicine, Dr. D. Y. Patil Medical College, Hospital, and Research Centre, Dr. D. Y. Patil Vidyapeeth (Deemed to be University), Pune, IND

**Keywords:** eye emergencies, eye infections, opthalmology ocular pathology, emergency medicine procedures, endopthalmitis, point-of-care ultrasound (pocus)

## Abstract

Acute and subacute vision loss often requires specialized evaluation in emergency departments. Endophthalmitis, a serious eye infection, can be challenging to diagnose early but is critical to identify due to its potential severity. This case report illustrates the use of ocular point-of-care ultrasound (POCUS) in diagnosing endophthalmitis in a 72-year-old male initially thought to have orbital cellulitis. Despite swollen and painful eyes hindering direct examination, the ocular POCUS examination revealed intraocular hyperechoic debris, indicating an intraocular infection suggestive of endophthalmitis. This timely diagnosis facilitated quick and appropriate treatment, highlighting the role of ocular POCUS in the emergency department for swift and accurate assessment.

## Introduction

Vision loss (acute/subacute) is frequently presented in the emergency department [1]. Identifying and evaluating these critical complaints can be challenging without the necessary equipment and expert knowledge [2]. A comprehensive assessment often necessitates a thorough physical examination, imaging studies, and consultation with an ophthalmologist, which may not always be quickly accessible in the emergency department [3,4]. Endophthalmitis is an infection of the eye caused by bacteria or fungi and is often overlooked in the early stage. This case report focuses on the significance of using ocular point-of-care ultrasound (POCUS) in a patient who presented with pain and gross swelling in one eye, which was then recognized as endophthalmitis using ocular POCUS. The patient's initial clinical evaluation favored a diagnosis of orbital cellulitis, which could have caused a delay in the timely and crucial detection of endophthalmitis. While ocular POCUS is generally used for various posterior eye conditions such as papilledema, retinal detachment, and vitreous detachment/hemorrhage, our case highlights its use in recognizing endophthalmitis when a physical examination is inconclusive [5-9]. This case report discusses the initial bedside ocular POCUS findings by the primary emergency medicine provider, leading to a rapid diagnosis and swift ocular intervention despite the limited physical examination due to severe pain and swelling of the affected left eye.

## Case presentation

**Figure 1:**
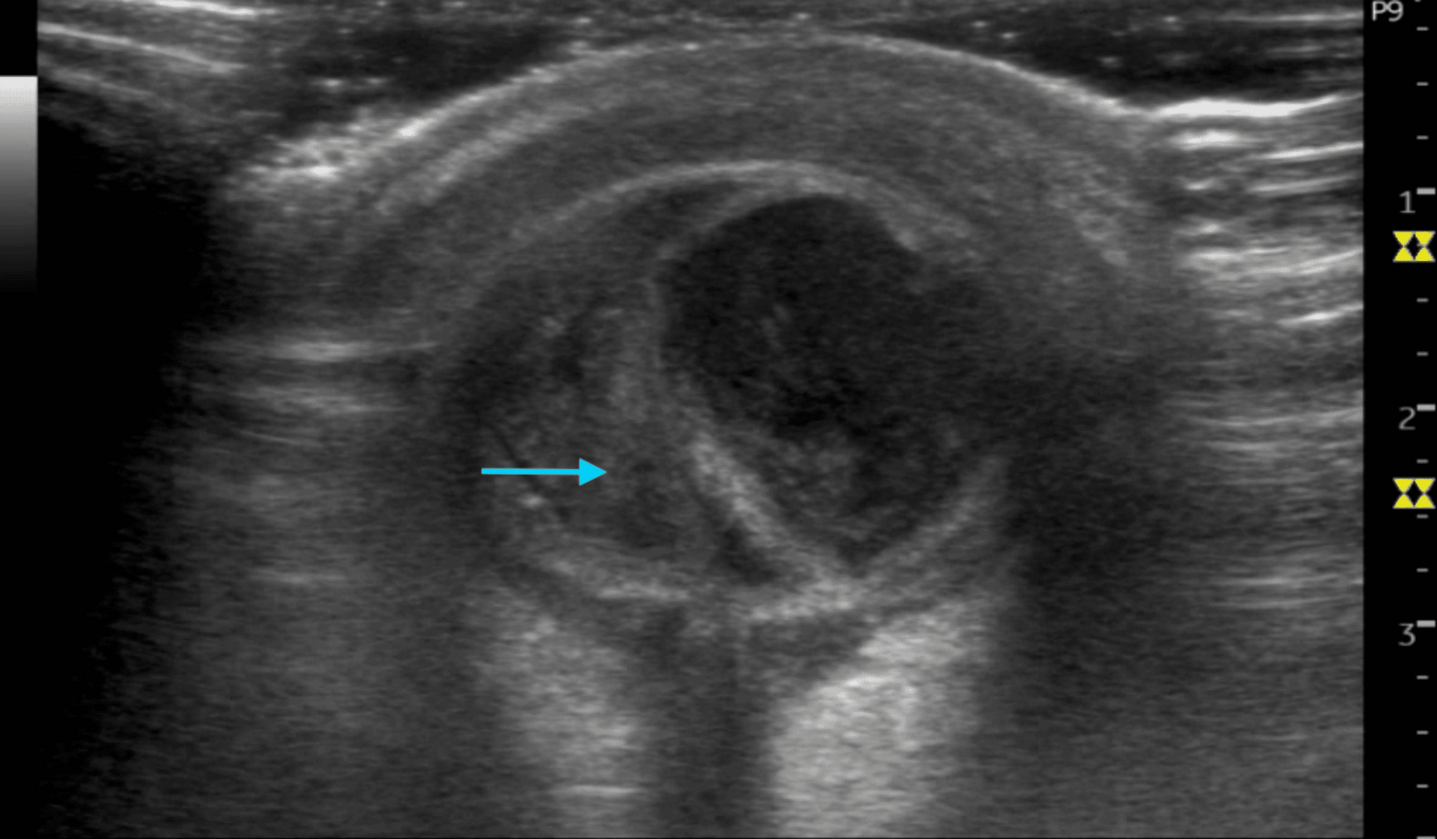
Intraocular debris seen on ocular POCUS of the left eye as depicted by an arrow. POCUS: point-of-care ultrasound

An elderly 72-year-old male without any prior medical history was brought to our emergency department with pain in the left eye and significant swelling persisting for seven to eight days. The swelling was acute in onset, gradually progressing, and accompanied by thick, whitish, and yellowish discharge. The swelling had exacerbated to the point that he was unable to open his left eye. On examination, there was pronounced periorbital edema and erythema in the upper and lower eyelids, along with a continuing mucopurulent discharge in the left eye. Eye opening revealed proptosis and significant chemosis, but the patient's pain, discomfort, and swelling did not allow for a complete ocular examination. Given the compromised visual clarity, discharge presence, and restricted direct examination due to chemosis, an ocular POCUS was conducted. For ocular POCUS examination, the patient was placed in a supine position, and a clear plastic (Tegaderm) was placed over the closed left eye to shield it, followed by the application of ultrasound gel. Using a Wipro GE LOGIQ P9 R3 portable ultrasound (Chicago, IL: GE HealthCare) equipped with an L3-12 RS linear transducer, images were obtained. Ocular POCUS revealed extensive and well-defined hyperechoic, mobile debris in the posterior chamber, along with additional hyperechoic debris observed in the lens (Figure 1).

**Figure 2:**
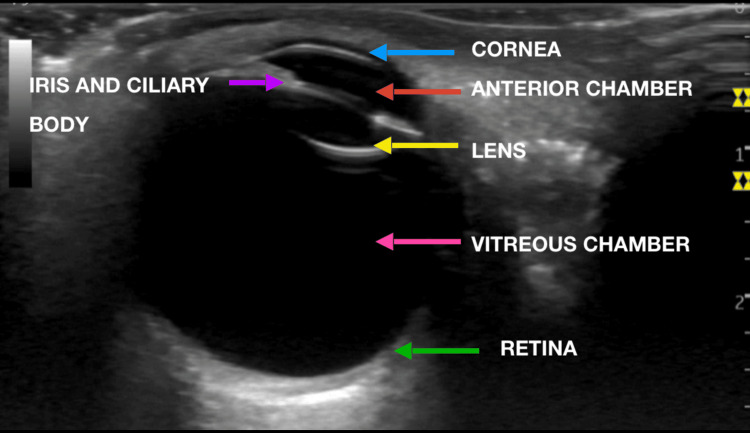
Ocular POCUS of the normal right eye. POCUS: point-of-care ultrasound

The findings in this study are strongly suggestive of endophthalmitis. For comparison, Figure 2 illustrates the ocular POCUS of a normal, healthy right eye. This ocular POCUS examination facilitated communication between the emergency department physician and the consulting ophthalmologist, expediting further management and planning. The patient was then transferred under the care of the consulting ophthalmologist for further management.

## Discussion

Endophthalmitis is a severe infection of the vitreous or aqueous humor within the eye. Due to the low presence of inflammatory cells and cytokines in the intraocular fluid, infections can develop insidiously and may remain unnoticed for a long period. Without prompt diagnosis, the infection can rapidly spread, causing irreversible damage to the eye and may ultimately lead to permanent vision loss [10]. Exogenous endophthalmitis, the more common form, occurs when microorganisms enter the eye from an external source and account for 40-80% of cases. Endogenous endophthalmitis, which accounts for up to 20% of cases, results from the hematogenous spread of infection to the eye [10]. In patients with endophthalmitis, ultrasound findings often include heterogeneous debris in the vitreous humor, choroidal thickening, posterior membrane detachment, choroidal detachment, and retinal detachment [11]. Interpreting these ultrasound findings is crucial for early diagnosis and treatment, thereby minimizing the risk of severe outcomes. In this case, the identification of debris in the vitreous humor on ultrasound, suggesting involvement of the posterior globe, raised substantial concerns regarding endophthalmitis.

Some studies have explored ultrasound characteristics seen with endophthalmitis. For example, Maneschg et al. noted detachment of the posterior vitreous membrane with vitreous opacifications in cases of endophthalmitis post-cataract surgery [12]. Kohanim et al. identified a mobile echogenic substance and thickening of the choroid and retina in infectious endophthalmitis [11]. Prognostically, Rachitskaya et al. suggested that significant vitreous membranes, dense vitreous opacities, and choroidal detachments with worse visual acuity are observed in cases of endophthalmitis [13]. Studies have highlighted the diagnostic complexities of endophthalmitis, frequently resulting in misdiagnosis or delayed diagnosis [14]. It has been established that endophthalmitis is associated with poor prognosis, leading to vision loss or necessitating surgical interventions like evisceration [15]. Although endophthalmitis is typically diagnosed through fundus examination and confirmed with culture tests, in our case, the patient's pronounced orbital swelling hindered a comprehensive fundoscopic examination. However, ultrasound findings were highly suggestive of endophthalmitis. Using ocular POCUS also reduced the time taken to reach the diagnosis significantly, as ocular POCUS requires very little time to conduct.

## Conclusions

Ocular POCUS is essential for managing ocular emergencies and providing rapid, accurate, and noninvasive evaluation of vision-threatening conditions. In this case, ocular POCUS easily identified endophthalmitis by revealing distinctive intraocular hyperechoic debris. This capability facilitates prompt and definitive treatment, complementing the role of ophthalmologists and reducing emergency department boarding time. While further research is needed to fully understand its benefits, and it is not yet the gold standard for diagnosis, incorporating ultrasound techniques into the diagnostic process enhances our ability to manage these conditions in the emergency department, ultimately improving patient care outcomes.
